# Randomized Pilot Study to Compare DCB-Based versus DST-Based Strategies for the Treatment of True or Complex Coronary Bifurcation Lesions

**DOI:** 10.31083/j.rcm2404099

**Published:** 2023-03-23

**Authors:** Dan Ke, Xi He, Canqiang Chen, Chaogui Lin, Yukun Luo, Lin Fan, Sumei Li, Xingchun Zheng, Lianglong Chen

**Affiliations:** ^1^Department of Cardiology, Fujian Medical University Union Hospital, 350001 Fuzhou, Fujian, China; ^2^Fujian Institute of Coronary Artery Disease, 350001 Fuzhou, Fujian, China

**Keywords:** percutaneous coronary intervention, drug-coated balloon, drug-eluting stent, true bifurcation lesion

## Abstract

**Background:**

Dual stenting technique (DST) is still mandatory for some true bifurcation lesions (BLs), but drug-coated balloon (DCB) alone may offer a new optional treatment with the potential benefits of fewer implants. However, procedural safety presents a concern when using DCB-only to treat true BLs. This study sought to explore the safety and efficacy of the DCB-only strategy for the treatment of true BLs.

**Methods:**

Sixty patients with TBLs were randomly assigned to be treated by a DCB-based strategy or DST-based strategy. All patients received angiographic follow-up scheduled after one-year and staged clinical follow-up. The primary endpoint was the one-year late lumen loss (LLL) and cumulative major cardiac adverse events (MACEs) composed of cardiac death (CD), target vessel myocardial infarction (TVMI), target lesion thrombosis (TVT), or target vessel/lesion revascularization (TLR/TVR). The secondary endpoint was the one-year minimal lumen diameter (MLD), diameter stenosis percentage (DSP) or binary restenosis (BRS), and each MACE component.

**Results:**

The baseline clinical and lesioncharacteristics were comparable with similar proportions (20.0% vs. 23.3%, *p = *1.000) of the complex BLs between the two groups. At the one-year follow-up, LLL was significantly lower in the DCB-based group (main-vessel: 0.05 ± 0.24 mm vs. 0.25 ± 0.35 mm, *p = *0.013; side-branch: –0.02 ± 0.19 mm vs. 0.11 ± 0.15 mm, *p = *0.005). MLD, DSP and TLR/TVR were comparable between the groups. The one-year cumulative MACE, all driven by TLR/TVR (6.7% vs. 13.3%, *p = *0.667), was low and similar without CD, TVMI or TVT in both groups.

**Conclusions:**

Compared to the DST strategy, the DCB- based strategy may be safe and effective in treatment of the selected true BLs.

**Clinical Trial Registration:**

Clinical registration number is ChiCTR1900024914.

## 1. Introduction

An ideal strategy of percutaneous coronary intervention (PCI) for bifurcation lesions (BLs) remains controversial. Provisional side-branch stenting (PSS) is recommended as the default treatment for most BLs [1, 2, 3], but main-branch (MB) stenting may cause carina or/and plaque shifting toward the ostial side-branch (SB), where acute compromise, dissection or occlusion, or chronic restenosis may occur, leading to poor outcomes [3, 4, 5, 6, 7, 8, 9]. As a result, dual stenting techniques (DSTs) with systematic stenting of both SB and MB, although technically complicated, are still mandatory for the treatment of true or complex BLs [1, 2, 3]. Nonetheless, compared to PSS, DSTs were not always associated with better long-term clinical outcomes as shown in previous studies [10, 11]. Therefore, exploringother novel techniques that can effectively avoid either PSS- or DST-associated weaknesses is necessary. With the advent of drug-coated balloons (DCB), a new DCB-only option has been attempted to treat BLs and has been shown to be technically feasible in a few pilot studies [12, 13], albeit existing worries about the procedural safety in the treatment of true or complex BLs. Fortunately, several newly-developed devices for lesion preparation along with more potent drugs for anti-thrombotic therapy may create a much safer milieu when using the DCB-only strategy for the treatment of BLs.

This study sought to explore the safety and efficacy of the DCB-based strategy in the treatment of true or complex BLs or to verify the concept of “bifurcation intervention with no implantation” (BINI) in these lesion subsets.

## 2. Methods

### 2.1 Study Design and Patient Selection

This is a single-center randomized pilot study. Patients with the following criteria were deemed eligible: (1) de novo true BLs (Medina type 1, 1, 1; 0, 1, 1; 1, 0, 1) and (2) SB ≥2.25 mm by visual estimation; Patients with the following criteria were excluded: (1) left main BLs, (2) other lesions requiring PCI in addition to the target BLs, and (3) lesions unsuitable for DCB treatment because of bifurcation or anatomy features (e.g., severe calcification, tortuous lesions, and wiring difficulty, etc.), (4) ST-elevation myocardial infarction (MI) within 48 h, (5) high bleeding risks, (6) allergy to any drugs needed, and (7) life expectancy <1 year.

**Fig. 1.**
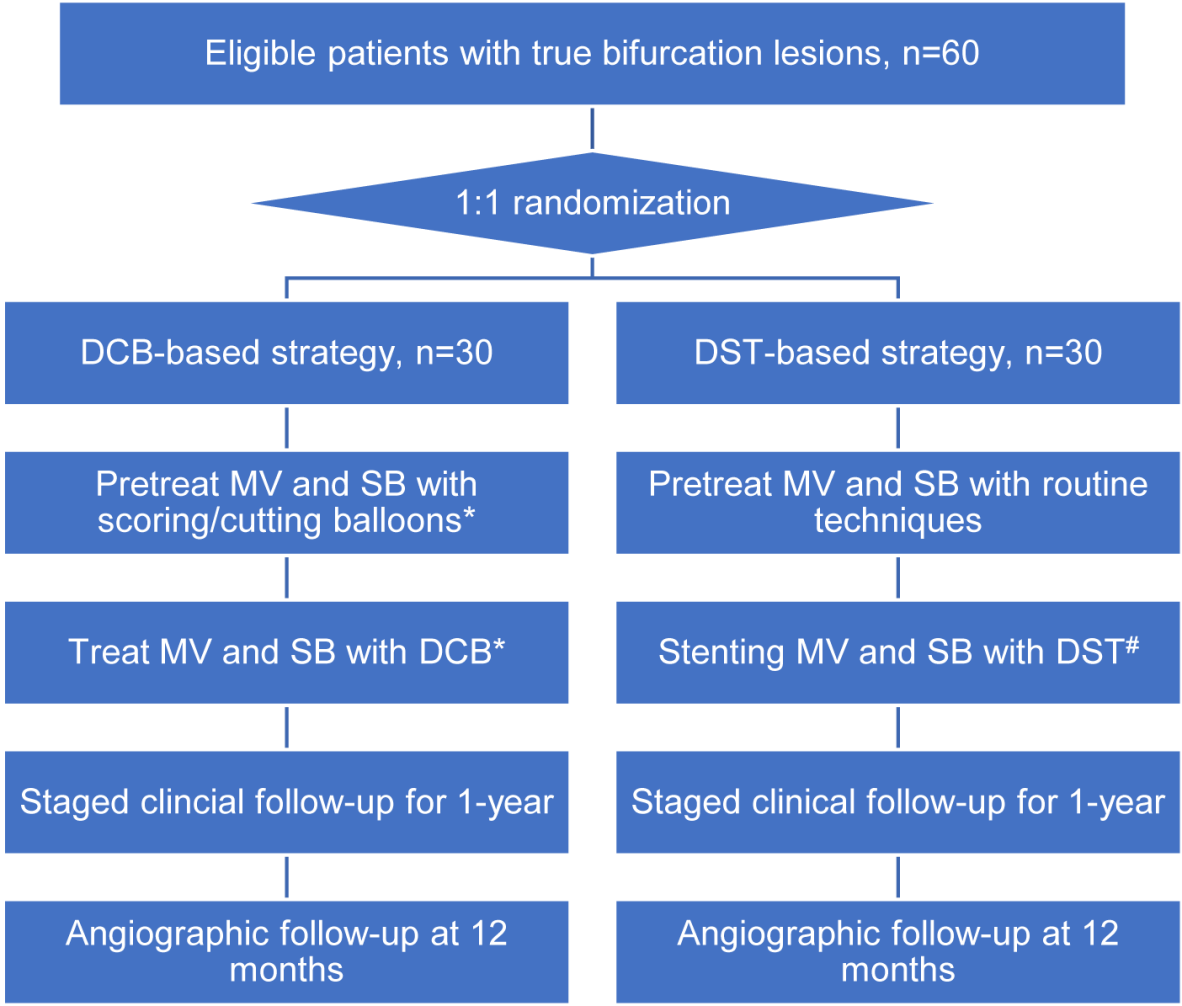
**Study Flowchart**. *, Bailout stenting of MV only or both MV and SB was allowable in lesion pretreatment or in DCB treatment if there were unacceptable results. ^#^, DST with using DK-crush, DK-culotte or T-stent was left at discretion of the operators. DCB, drug-coated balloon; DST, dual-stenting techniques; MV, main-vessel; SB, side-branch.

From Feb. 2019 to Feb. 2021, a total of 60 patients were randomized at a 1:1 ratio to receive either a DCB-based strategy or a DST-based strategy for BL intervention and then scheduled follow-up (Fig. 1). The protocol was approved by the Ethics Committee of Fujian Medical University Union Hospital (Supplementary Approval File No 2019KY035). All patients gave written informed consent.

### 2.2 Procedures

**Fig. 2.**
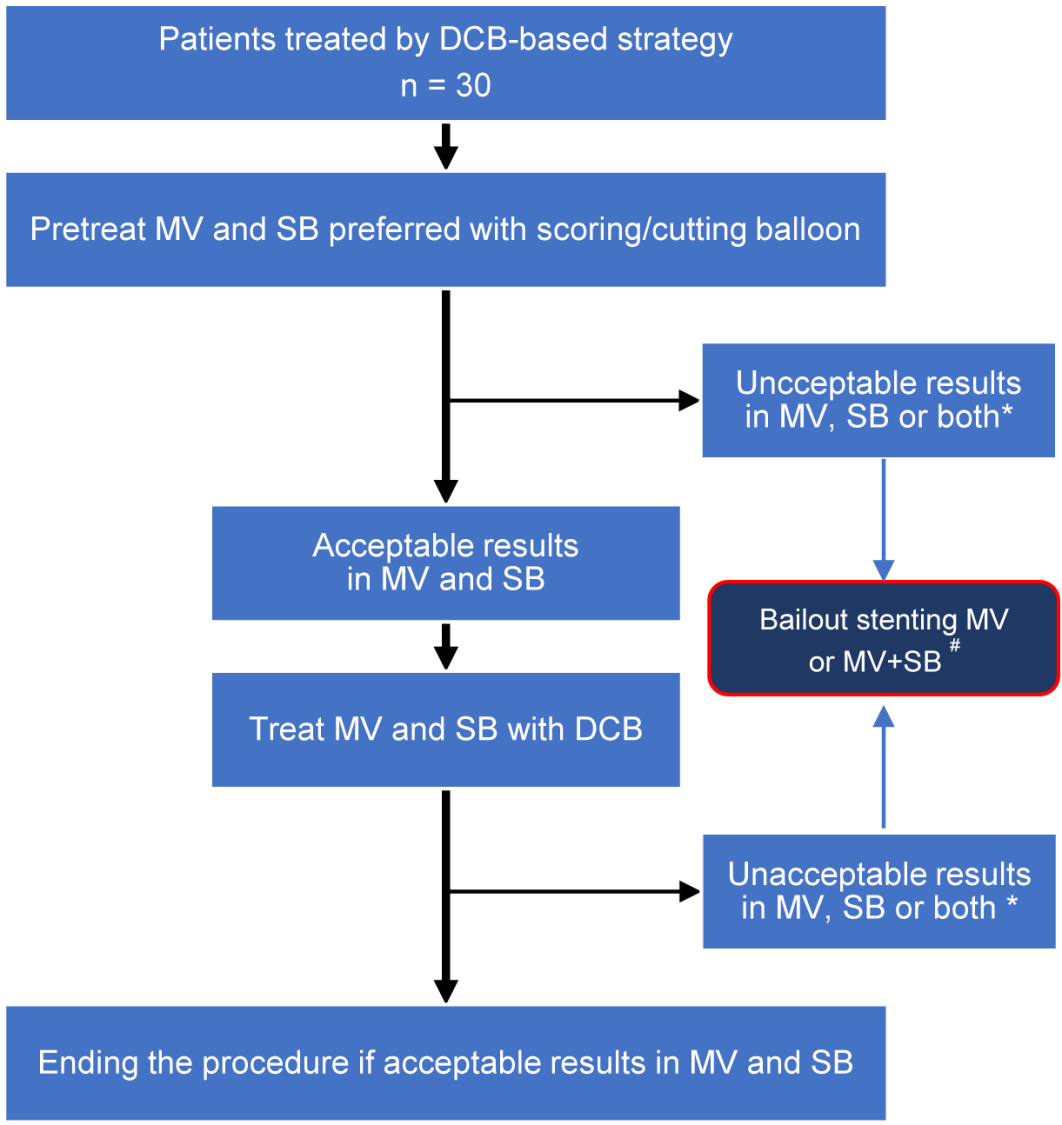
** The Procedural Steps of DCB-based Strategy**. *, Unacceptable results were defined as any of residual stenosis >30% or flow-limiting dissection either in MV, SB or both. ^#^, Bailout stenting of MV only or both MV and SB was left at discretion of the operators. DCB, drug-coated balloon; MV, main-vessel; SB, side-branch.

**DCB-based strategy**: This technique is a combined approach, characterized by DCB-centered angioplasty, optimal lesion pretreatment, and allowable use of bailout stenting and GP IIb/IIIa inhibitors, to ensure procedural safety. The key steps (Fig. 2) are briefly described below: (1) Scoring or cutting balloons was preferred for lesion preparation, and pre-dilating with smaller plain balloons for subsequent passage of scoring or cutting balloons or post-dilating with larger non-compliant balloons for achievement of an optimal lumen was allowable. (2) After optimal lesion preparation of the MV and SB, DCB angioplasty was performed on the SB and then the MV, and final kissing dilation was at the discretion of the operators. (3) The diameter of proximal and distal MB was averaged as the reference vessel diameter (RVD) of MV, a balloon to RVD ratio of ≈1.0 was adopted in the final lesion preparation and DCB angioplasty. (4) Bailout stenting for MV or MV+SB was allowable if unacceptable results [14, 15, 16] were obtained in lesion preparation or DCB angioplasty stage.

**DST-based strategy**: One of the DSTs (DK-crush, DK-culotte or T-stenting) may be selected and should be completed according to the standards of various DSTs [2, 3].

### 2.3 Materials

The DCB was a paclitaxel/iopromide matrix coating balloon (SeQuent® Please, B. Braun Melsungen AG, Germany). All stents were the 2nd generation drug-eluting stents, including ${\mathrm{Resolute}}^{\text{TM}}$ (Medtronic, Minneapolis, MN, USA), ${\mathrm{Xience}}^{\text{TM}}$ (Abbott Vascular, Santa Clara, CA, USA), Firebird-$2^{\text{TM}}$ (Microport, Shanghai, China), and ${\mathrm{Excel}}^{\text{TM}}$ (JW, Shandong, China).

### 2.4 Medications

All patients received pretreatment with aspirin and P2Y12 antagonists of clopidogrel or ticagrelor with a loading dose as indicated. Intra-procedural heparin (70–100 U/kg) was intravenously injected with a supplemented bolus of 1000 U given per hour to maintain an activated clotting time of 250–300 seconds. Peri-procedural use of glycoprotein IIb/IIIa inhibitors was allowable at the operator’s discretion. Dual anti-platelet therapy with aspirin plus clopidogrel or ticagrelor (preferred) was maintained for one year for both strategies, followed by indefinite single anti-platelet therapy (aspirin, clopidogrel or ticagrelor).

### 2.5 Quantitative Coronary Angiography

Coronary angiography (CAG) was performed pre-procedurally, post-procedurally, and at follow-up after intracoronary injection of 200 μg nitroglycerin.

For quantitative coronary angiographic analysis (QCA), the bifurcation was simply segmented into: (1) MV, the segment from the proximal to distal end treated by stents or DCBs; and (2) SB, the segment from the carina to distal end treated by stents or DCBs. The reference vessel diameter (RVD) of the MV was the averaged diameter of the proximal and distal MB, and the minimal lumen diameter (MLD) was directly measured at the narrowest site. The diameter stenosis percent (DSP) was calculated by (RVD-MLD) / RVD × 100%, and the late lumen loss (LLL) was calculated as the post-procedural MLD — follow-up MLD. Binary restenosis (BRS) was defined as DSP >50%.

### 2.6 Follow-Up

Clinical data were collected during the hospital stay and by hospital visit or telephone contact at 1, 3, 6, 9, and 12 months after discharge and afterward annually thereafter. Follow-up CAG was scheduled at 12 ± 1 months post-procedurally.

### 2.7 Events and Definitions 

All deaths were deemed cardiogenic unless there was clear evidence of non-cardiac causes. Peri-procedural MI (within 48 h) was defined as: I. a creatine kinase-MB (CK-MB) >10 or troponin >70 × the upper reference limit (URL), or II. a CK-MB >5 or troponin >35 × URL plus either: (1) new pathological Q waves in ≥2 contiguous leads or new left bundle branch block; (2) angiographically documented graft or coronary artery occlusion or new severe stenosis with thrombosis; (3) imaging evidence of new loss of viable myocardium; or (4) new regional wall motion abnormality. In non-ST elevation MI (NSTEMI) patients with elevated pre-procedural biomarkers in whom the levels were stable (≤20% variation) or falling, peri-procedural MI could be diagnosed when the post-procedural biomarkers rise by >20% along with the criteria similar to the aforementioned Definition II. Spontaneous MI (after 48 h) was defined as a clinical syndrome consistent with MI along with a CK-MB or troponin >1 × URL and new ST-segment elevation or depression or other findings as described above. All MI were considered target vessel myocardial infarction (TVMI) unless there was clear evidence attributable to a non-target vessel. Clinically driven TLR/TVR was defined as typical angina pectoris or confirmed ischemia referable to the target lesion/vessel requiring urgent or selective repeat PCI or coronary artery bypass graft. TVST was determined according to the ARC classification [17]. The major cardiac adverse event (MACE) is defined as a composite of cardiac death, TVMI, target vessel thrombosis (TVT) or ischemia-driven target vessel/lesion revascularization (TLR/TVR).

### 2.8 Outcomes

The primary outcomes were the peri-procedural MACE, one-year cumulative MACE and angiographic LLL. The secondary outcomes were each component of MACE, MLD and BRS.

### 2.9 Statistical Analysis

Data were expressed as the mean ± SD for continuous variables or as frequency (%) for discrete or categorical variables. To compare differences between groups, Student’s *t test *was used for continuous variables, and the chi-square or Fisher’s exact test was used for the discrete variables. A *p* value of <0.05 was considered statistically significant.

All analyses were performed with SSPS (version 20.0, IBM Corp., Chicago, IL, USA).

## 3. Results

### 3.1 Baseline Clinical and Lesion Characteristics

The clinical characteristics were balanced between the two groups (Table 1). The use of aspirin, clopidogrel or ticargrelor was similar in the groups regardless of the more frequent use of ticargrelor or less frequent use of clopidogrel in the DCB-based group.

**Table 1.** **Baseline clinical and lesion characteristics**.

		DCB-based strategy (n = 30)	DST-based strategy (n = 30)	*p* value
Age, years		58.6 ± 10.3	61.4 ± 8.5	0.268
Gender, male (%)		26 (86.7)	25 (83.3)	1.000
Hypertension, n (%)		18 (60.0)	21 (70.0)	0.588
Hypercholesteremia, n (%)		22 (73.3)	20 (66.7)	0.778
Diabetes mellitus, n (%)		9 (30.0)	11 (36.7)	0.784
Current smoker, n (%)		16 (53.3)	19 (63.3)	0.600
History of PCI, n (%)		5 (16.7)	4 (13.3)	1.000
Previous MI, n (%)		3 (10.0)	2 (6.7)	1.000
LVEF, %		62.48 ± 7.76	64.00 ± 11.36	0.199
Clinical presentation, n (%)				
	NSTEMI	4 (13.3)	6 (20.0)	0.729
	Unstable angina	10 (33.3)	10 (33.3)	1.000
	Stable angina	16 (53.3)	14 (46.7)	0.796
Antiplatelet therapy, n (%)				
	Aspirin	30 (100.0)	30 (100.0)	1.000
	Clopidogrel	14 (46.7)	16 (53.3)	0.796
	Ticargrelor	16 (53.3)	14 (46.7)	0.796
Bifurcation anatomy, n (%)				
	Y-type (distal angle <70°)	24 (80.0)	25 (83.3)	1.000
	T-type (distal angle ≥70°)	6 (20.0)	5 (16.7)	1.000
Lesion location, n (%)				
	LAD	18 (60.0)	17 (56.7)	1.000
	LCX	8 (26.7)	7 (23.3)	1.000
	RCA	4 (13.3)	6 (20.0)	0.729
Medina classification, n (%)				
	1, 1, 1	15 (50.0)	14 (46.7)	1.000
	0, 1, 1	11 (36.7)	11 (36.7)	1.000
	1, 0, 1	4 (13.3)	5 (16.7)	1.000
Lesion complexity*, n (%)				
	Complex	6 (20.0)	7 (23.3)	1.000
	Simple	24 (80.0)	23 (76.7)	1.000
Lesion length, mm				
	MV/MB	21.97 ± 6.98	22.77 ± 9.02	0.702
	SB	13.0 ± 5.02	12.7 ± 3.20	0.807
Diameter stenosis, %				
	MV/MB	79.0 ± 7.81	80.33 ± 8.50	0.530
	SB	63.5 ± 14.09	61.67 ± 13.60	0.610

DCB, drug-coated balloon; DST, dual stenting technique; LAD, left anterior descending artery; LCX, left circumflex artery; LVEF, left ventricular ejection fraction; MB, main-branch; MI, myocardial infarction; MV, main-vessel; NSTEMI, non–ST-segment elevation myocardial infarction; PCI, percutaneous coronary intervention; RCA, right coronary artery; SB, side-branch. Abbreviation was similar in the following tables unless otherwise indicated. *, lesion complexity was determined by the Definition criteria.

No difference was observed in lesion features between the two groups, especially in bifurcation angulation, branch stenotic severity and lesion length between the two groups, and the proportion of true BLs (100% vs. 100%) and complex BLs (20.0% vs. 23.3%) were similar between the DCB- and the DST-based groups (Table 1).

### 3.2 Procedural Characteristics

**Fig. 3.**
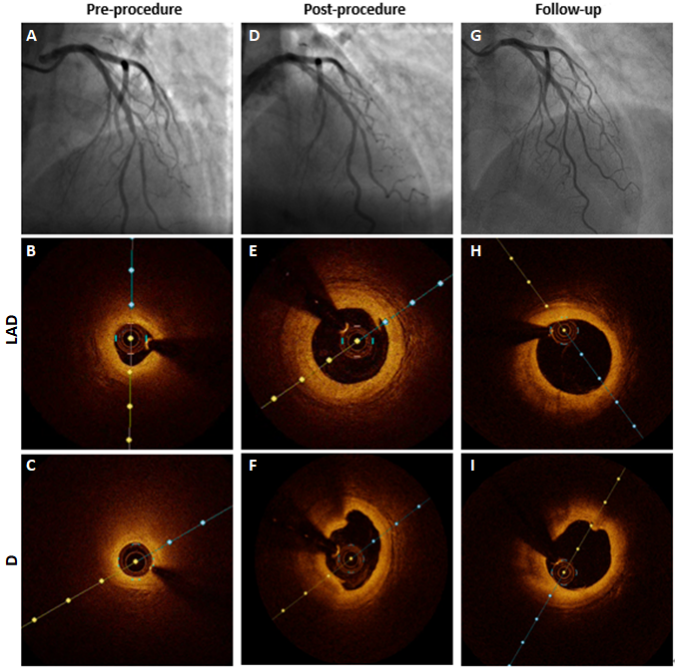
** Healing of non-flow-limiting dissection during follow-up**. CAG and OCT show a typical true BL affected LAD-D pre-procedurally (A,B,C) with lipid-rich plaque in LAD (B) and D (C), several minor dissections observed post-procedurally (D,E,F) in LAD (E) and D (F) and no more dissections found at 1-year follow-up (G,H,I) in the corresponding site. CAG, coronary angiography; D, diagonal artery; LAD, left anterior descending artery; OCT, optical coherence tomography; BL, bifurcation lesion.

Procedural data are shown in Table 2. As the DCB-based strategy is a preset combined approach, there was more frequent use of scoring or cutting balloons (MV: 50% vs. 6.7%, *p* = 0.000; SB: 63.3% vs. 6.7%, *p* = 0.000) and GP IIb/IIIa inhibitors (60% vs. 6.7%, *p <* 0.001) were observed in the DCB-based group. The length of stenting or DCB angioplasty for both branches was comparable between the groups with less final kissing dilation (26.7% vs. 100%, *p* = 0.000) in the DCB-based group. Although dissection < Type C occurred more frequently in the DCB-based group (MV: 26.7% vs. 3.3%, *p* = 0.030; SB: 36.7% vs. 6.7%, *p* = 0.012), there was neither flow-limiting dissection and the associated events nor requirement of bailout stenting during the procedures were noted in the DCB-based group. Optical coherence tomography (OCT) showed that these dissections were minor with an arc <60°, a length of <2 mm and limited to the intima (Fig. 3). The rate of immediate angiographic success defined by residual stenosis <20% was lower in both branches (MV: 46.7% vs. 96.7%, *p *< 0.001; SB: 33.3% vs. 80.0%, *p *< 0.001) in the DCB-based group, but the rate of immediate residual stenosis >30% was low and similar in both branches (MV: 6.7% vs. 3.3%, *p* = 1.000; SB: 10.0% vs. 3.3%, *p* = 0.605) between the groups.

**Table 2.** **Procedural characteristics**.

		DCB-based strategy (n = 30)	DST-based strategy (n = 30)	*p *value
Trans-radial approach, n (%)		30 (100.0)	30 (100.0)	1.000
MV/MB preparation, n (%)		30 (100.0)	30 (100.0)	1.000
	Scoring/Cutting balloon	24 (80.0)	2 (6.70)	0.000
	Non-complaint balloon	24 (80.0)	30 (100.0)	0.031
SB preparation, n (%)		30 (100)	27 (90.0)	1.000
	Scoring/Cutting balloon	19 (63.3)	2 (6.70)	0.000
	Non-complaint balloon	18 (60.0)	30 (100.0)	0.000
DCB angioplasty, n (%)		30 (100)	-	N/A
	MV/MB	30 (100)	-	N/A
	SB	30 (100)	-	N/A
Length of stent or DCB, mm				
	MV/MB	27.67 ± 6.91	26.87 ± 9.17	0.704
	SB	18.83 ± 4.68	17.13 ± 3.46	0.115
Final kissing dilation, n (%)		8 (26.7)	30 (100.0)	0.000
Residual stenosis >20%, n (%)				
	MV/MB	16 (53.3)	1 (3.3)	<0.001
	SB	20 (66.7)	6 (20.0)	0.001
Residual stenosis >30%, n (%)				
	MV/MB	2 (6.70)	1 (3.3)	1.000
	SB	3 (10.0)	1 (3.3)	0.605
TIMI flow grade <3, n (%)				
	MV/MB	0 (0.0)	0 (0.0)	1.000
	SB	0 (0.0)	0 (0.0)	1.000
Dissection ≥type C, n (%)				
	MV/MB	0 (0.0)	0 (0.0)	1.000
	SB	0 (0.0)	0 (0.0)	1.000
Dissection <type C, n (%)				
	MV/MB	8 (26.7)	1 (3.3)	0.030
	SB	11 (36.7)	2 (6.7)	0.012
Stenting or Bail-out stenting*, n (%)				
	MV/MB	0 (0.0)	30 (100.0)	<0.001
	SB	0 (0.0)	30 (100.0)	<0.001
Angiographic ${\mathrm{success}}^{\#}$, n (%)				
	MV/MB	14 (46.7)	29 (96.7)	<0.001
	SB	10 (33.3)	24 (80.0)	<0.001
Use of GP IIb/IIIa inhibitor, n (%)		18 (60.0)	2 (6.7)	<0.001

DCB, drug-coated balloon; DST, dual stenting technique; MB, main-branch; MV, main-vessel; SB, side-branch; TIMI, thrombolysis in myocardial infarction; GP, glycoprotein. *, For DCB-based strategy, bailout stenting of MV, or MV+SB was indicated if any of acute occlusion or flow-limiting dissection in the stage of lesion preparation or DCB angioplasty; while for DST-based strategy, both branches were stented per protocol in all patients. ^#^, Angiographic success was defined as residual stenosis <20% without flow-limiting dissection or bailout stenting in both branches.

### 3.3 Angiographic Outcomes

QCA data are listed in Table 3. Compared to the DST-based group, the DCB-based group had a reduced LLL in both branches (MB: 0.05 ± 0.24 mm vs. 0.25 ± 0.35 mm, *p* = 0.013; SB: –0.02 ± 0.19 mm vs. 0.11 ± 0.15 mm, *p* = 0.005). In line with LLL, there were similar MLD and DSP and BRS were observed in both branches between the groups.

**Table 3.** **Quantitative coronary angiographic analysis**.

			DCB-based strategy (n = 30)	DST-based strategy (n = 30)	*p* value
Pre-procedure					
	RVD, mm				
		MV	2.97 ± 0.34	2.93 ± 0.41	0.719
		SB	2.37 ± 0.19	2.33 ± 0.19	0.504
	MLD, mm				
		MV	0.62 ± 0.25	0.57 ± 0.25	0.400
		SB	0.89 ± 0.33	0.89 ± 0.32	0.949
	DSP, %				
		MV	79.00 ± 7.81	80.33 ± 8.50	0.530
		SB	63.50 ± 14.09	61.67 ± 13.60	0.610
	LL, mm				
		MV	21.97 ± 6.98	22.77 ± 9.02	0.702
		SB	13.00 ± 5.02	12.70 ± 3.20	0.807
Post-procedure					
	RVD, mm				
		MV	2.95 ± 0.35	2.88 ± 0.43	0.492
		SB	2.32 ± 0.20	2.30 ± 0.18	0.690
	MLD, mm				
		MV	2.26 ± 0.41	2.60 ± 0.39	0.001
		SB	1.77 ± 0.31	2.02 ± 0.37	0.007
	DSP, %				
		MV	23.00 ± 11.19	9.00 ± 8.85	0.001
		SB	23.67 ± 10.80	12.50 ± 12.09	<0.001
Follow-up					
	RVD, mm				
		MV	2.97 ± 0.44	2.86 ± 0.36	0.295
		SB	2.34 ± 0.20	2.29 ± 0.18	0.368
	MLD, mm				
		MV	2.21 ± 0.35	2.35 ± 0.56	0.233
		SB	1.80 ± 0.33	1.91 ± 0.41	0.184
	DSP, %				
		MV	24.26 ± 13.90	17.00 ± 21.21	0.122
		SB	23.20 ± 11.41	16.72 ± 15.45	0.07
	LLL, mm				
		MV	0.05 ± 0.24	0.25 ± 0.35	0.013
		SB	–0.02 ± 0.19	0.11 ± 0.15	0.005
	BRS, n (%)				
		MV	2 (6.7)	2 (6.7)	1.000
		SB	0 (0.0)	2 (6.7)	0.472

DCB, drug-coated balloon; DST, dual stenting technique; BRS, binary restenosis; DSP, diameter stenosis percent; LL, lesion length; LLL, late lumen loss; MV, main-vessel; MLD, minimal lumen diameter; RVD, reference vessel diameter; SB, side-branch.

### 3.4 Clinical Outcomes

No patients were lost to follow-up. The rates of peri-procedural MACEs (0.0% vs. 0.0%, *p* = 1.000) and one-year cumulative MACEs driven all by TLR/TVR (6.70% vs. 13.30%, *p* = 0.667) were similar without death and TVT between the DCB- and the DST-based groups (Table 4). The occurrence of post-procedural troponin elevation of ≥5 × URL was similar between the groups (16.7% vs. 20.0%, *p* = 1.000).

**Table 4.** **MACE and its components**.

		DCB-based strategy (n = 30)	DST-based strategy (n = 30)	*p* value
Peri-procedural MACE, n (%)		0 (0.0)	0 (0.0)	1.000
Death		0 (0.0)	0 (0.0)	1.000
	Non-Cardiac	0 (0.0)	0 (0.0)	1.000
	Cardiac	0 (0.0)	0 (0.0)	1.000
TVMI		0 (0.0)	0 (0.0)	1.000
	Peri-procedural MI	0 (0.0)	0 (0.0)	1.000
	Spontaneous MI	0 (0.0)	0 (0.0)	1.000
TVT		0 (0.0)	0 (0.0)	1.000
TLR/TVR		0 (0.0)	0 (0.0)	1.000
1-year Cumulative MACE, n (%)		2 (6.70)	4 (13.3)	0.667
Death		0 (0.0)	0 (0.0)	1.000
	Non-Cardiac	0 (0.0)	0 (0.0)	1.000
	Cardiac	0 (0.0)	0 (0.0)	1.000
TVMI				
	Peri-procedural MI	0 (0.0)	0 (0.0)	1.000
	Spontaneous MI	0 (0.0)	0 (0.0)	1.000
TVT		0 (0.0)	0 (0.0)	1.000
TLR/TVR		2 (6.70)	4 (13.3)	0.667

DCB, drug-coated balloon; DST, dual stenting technique; MI, myocardial infarction; MACE, major cardiac adverse event; TLR/TVR, target vessel/lesion revascularization; TVMI, target vessel myocardial infarction; TVT, target vessel thrombosis.

## 4. Discussion

This study was the first to randomly compare the DCB-based strategy versus the DST-based strategy in the treatment of true BLs with partial complex BLs. The major findings showed that the DCB-based strategy was associated with less LLL or even negative LLL, similar peri-procedural safety in terms of neither flow-limiting dissection and the associated events nor requirement of intra-procedural bailout stenting, and similar one-year cumulative MACEs compared to the DST-based strategy.

The introduction of DCBs offers new options to simply bifurcation intervention. Two approaches of DCB angioplasty approaches are employed for BLs [15, 16]: (1) the PSS strategy with DES implantation for MB and DCB angioplasty for SB; and (2) the DCB-only strategy for either MB or SB, or both, the so-called BINI. The updated guidelines and consensuses recommend PSS as the default treatment for the majority of BLs [1, 2, 3]. In this setting, when SB treatment is indicated, angioplasty with DCB, which can locally deliver anti-proliferative agent into the vascular wall, may be preferable to angioplasty with plain balloon alone. In previous observational studies, better SB results were achieved by adding DCB angioplasty to SB when using the PSS strategy [18, 19, 20]. For the DCB-only strategy for BLs or BINI, two randomized pilot trials comparing DCB-only versus plain balloon-only for the treatment of de novo BLs (Medina class 0,1,1) showed lower rates of restenosis and TLR in the DCB-only approach [12, 13]. Additionally, the DCB-only strategy for MB was often adequate and supported by the fact that ostial SB lesions might exhibit positive remodeling [21]. However, the DCB-only strategy for BLs, although been proposed and practiced clinically, has not been well tested against the standard approach of DSTs especially in the treatment of the true or complex BLs. The DCB-only strategy for BL intervention presents two major concerns: peri-procedural safety and long-term efficacy.

As characterized by BINI, the DCB-only strategy may introduce procedure-related risks such as acute dissection, thrombosis, occlusion, MI and likely fatal events [15, 16], so that bailout stenting may be required for severe dissection or occlusion as previously reported in 1–22% cases [16]. For sake of procedural safety, the severe calcified and tortuous lesions were excluded in our study. Crucially, this study adopted the combined lesion preparation for DCB angioplasty. In the DCB-based group, a scoring/cutting balloon for lesion preparation was used in most BLs (80% for MV, 63.3% for SB), DCB angioplasty in all BLs (100% for MV and SB), and GP IIb/IIIa inhibitor for enhancing anti-thrombosis in 60% of patients, all of which represent the typical DCB-centered combined strategy described above. The optimized lesion preparation and the proper selection of the lesions may explain no bailout stenting in our study. As shown in our study, although all included lesions were true BLs with 20% complex BLs, there was no requirement for intra-procedural bailout stenting because of flow-limiting dissection and the associated events in the DCB-based group regardless of the frequent occurrence of non-flow-limiting dissection during the procedure. Thus, the DCB-based strategy for the true or complex BLs may be technically feasible and procedurally safe. Moreover, although the variables of MLD, residual stenosis or angiographic success immediately after the procedures in the DCB-based group were inferior to those in the DST-based group, these variables and cumulative MACEs at the one-year follow-up were similar between the two groups, suggesting that the DCB-based strategy for the true or complex BLs may be similarly efficacious as compared with the DST-based strategy.

Surprisingly, at the 1-year follow-up, as shown in Fig. 3, all intra-procedural dissections (<Type C) eventually healed without adverse events, and less LLL was noted even with negative LLL (positive remodeling) in the DCB-based treatment, all of which, similar to the findings in previous studies [22, 23, 24, 25, 26, 27, 28], likely reflect a natural healing process after DCB angioplasty. This healing process can well explain the phenomenon whereby immediate suboptimal results become optimal at the 1-year follow-up.

Despite its randomized controlled design, our study still has several limitations. First of all, the single center pilot study with a small sample size might limit the generalizability of the results and conclusion. Second, the enrolled patients were not all comer given the exclusion of lesions unsuitable for DCB or PCI treatment such as severe calcified or tortuous lesions, left main bifurcations and so on, were excluded. Third, the one-year clinical and angiographic follow-up were not long enough to determine the long-term clinical outcomes. Fourth, lesions with thrombus in NSTEMI patients may contribute to lumen improvement at the 1-year follow-up in the DCB-based group. Therefore, a large-scale randomized trial is warranted to further validate the results.

## 5. Conclusions

This study demonstrated that compared to the DST-based strategy, the DCB-based strategy was associated with less LLL, similar procedural safety and a similarly low rate of one-year MACEs, thereby suggesting that the DCB-based approach may be a reasonable option in the treatment of the true or complex BLs given proper selection and preparation of this lesion subset.

## Data Availability

All data generated or analyzed during this study are included in this published article.

## Abbreviations

BL, coronary bifurcation lesion; DCB, drug-coated balloon; DES, drug-eluting stent; DST, Dual stenting technique; PCI, percutaneous coronary intervention; PSS, provisional side-branch stenting.
